# Riluzole partially restores RNA polymerase III complex assembly in cells expressing the leukodystrophy-causative variant POLR3B R103H

**DOI:** 10.1186/s13041-022-00974-z

**Published:** 2022-11-30

**Authors:** Maxime Pinard, Samaneh Dastpeyman, Christian Poitras, Geneviève Bernard, Marie-Soleil Gauthier, Benoit Coulombe

**Affiliations:** 1 Translational Proteomics Laboratory, Institut de Recherches Cliniques de Montréal, Montréal, Québec Canada; 2 Child Health and Human Development Program, Research Institute of the McGill University Health Centre, Montreal, Canada; 3 Department of Neurology and Neurosurgery, McGill University, Montreal, Canada; 4 Department of Human Genetics, McGill University, Montreal, Canada; 5 Department of Pediatrics, McGill University, Montreal, Canada; 6 Department of Specialized Medicine, Division of Medical Genetics, McGill University Health Center, Montreal, Canada; 7 Department of Biochemistry and Molecular Medicine, Université de Montréal, Montréal, Québec Canada

**Keywords:** RNA polymerase III, Protein complex assembly, POLR3-related leukodystrophy, 4H leukodystrophy, Riluzole, Drug repurposing, PAQosome, Mass spectrometry

## Abstract

The mechanism of assembly of RNA polymerase III (Pol III), the 17-subunit enzyme that synthesizes tRNAs, 5 S rRNA, and other small-nuclear (sn) RNAs in eukaryotes, is not clearly understood. The recent discovery of the HSP90 co-chaperone PAQosome (Particle for Arrangement of Quaternary structure) revealed a function for this machinery in the biogenesis of nuclear RNA polymerases. However, the connection between Pol III subunits and the PAQosome during the assembly process remains unexplored. Here, we report the development of a mass spectrometry-based assay that allows the characterization of Pol III assembly. This assay was used to dissect the stages of Pol III assembly, to start defining the function of the PAQosome in this process, to dissect the assembly defects driven by the leukodystrophy-causative R103H substitution in POLR3B, and to discover that riluzole, an FDA-approved drug for alleviation of ALS symptoms, partly corrects these assembly defects. Together, these results shed new light on the mechanism and regulation of human nuclear Pol III biogenesis.

**Supplementary Information:**

The online version contains supplementary material available at 10.1186/s13041-022-00974-z.

## Introduction

Pol III is one of the three eukaryotic nuclear RNA polymerases, the two others being RNA polymerase I (Pol I) and RNA Polymerase II (Pol II). Pol III synthetizes small non-coding RNAs, such as tRNAs, 5 S RNA, 7SK RNA and U6 RNA, that are involved in the regulation of essential cellular processes, including transcription, RNA processing, ribosome biogenesis and translation [1]. Pol I synthesizes large rRNAs [2] while Pol II synthesizes all mRNAs [3]. Each polymerase possesses its own set of accessory factors that are required to transcribe their specific set of genes. Pol III, for which the human crystal structure was elucidated recently [4, 5], is comprised of 17 core subunits, two being shared uniquely with Pol I and five others shared by the three Pols. We and others have shown that biogenesis of nuclear RNA polymerases involves a novel HSP90 co-chaperone complex named the PAQosome (Particle for Arrangement of Quaternary structure) [6–8].

The PAQosome, also known as R2TP/prefoldin-like (PFDL), is a 12-subunit co-chaperone complex responsible for the biogenesis of a vast number of multi-protein complexes in humans [8, 9]. It was first observed in association with subunits of all three nuclear RNA polymerases and most likely promotes their cytoplasmic assembly before translocation of mature complexes into the nucleus [6, 7]. Its *Saccharomyces cerevisiae* counterpart, which lacks the PFDL module, was first identified based on its association with HSP90 [10]. Client specificity by the PAQosome was shown to involve at least three distinct mechanisms: (1) the use of specific adaptors that connect client and PAQosome subunits [11, 12], (2) the existence of alternative PAQosome complexes that incorporate homologous subunits of RPAP3 and PIH1D1 including a testis-specific version of the PAQosome that interacts with a unique set of clients [13, 14], and (3) phosphorylation and dephosphorylation of some PAQosome subunits that induce interaction with its clients [15]. In addition to its role in RNA polymerases assembly, the PAQosome was shown to interact with snoRNP complex [16–18] and to affect the cellular response to stress through stabilization of phosphatidylinositol 3-kinase-related kinases (PIKK) [19] and promote their assembly into complexes including mTORC1 [20, 21], SMG-1 [20] the nonsense-mediated mRNA decay [22] or the DNA damage response ATR and ATM [19]. It was also shown to participate in the biogenesis of spliceosome component U4 and U5 snRNPs [11] and recently, our group reported that the PAQosome is involved in pre-ribosomal complex formation [15].

Leukodystrophies are genetic diseases of the cerebral white matter that are most commonly neurodegenerative in nature [23]. A subset of leukodystrophies named RNA polymerase III-related leukodystrophy or 4 H (Hypomyelination, Hypodontia and Hypogonadotropic Hypogonadism) leukodystrophy [24] (MIM 607,694, 614,381) was found to be caused by biallelic pathogenic variants in genes encoding specific subunits of the enzyme Pol III, such as POLR3A [25–27], POLR3B [28], POLR3K [29] and POLR1C [30]. Affinity purification experiment coupled with mass spectrometry (AP-MS) performed in cultured cells as a model system and in which wild-type (WT) and mutated subunits were compared, revealed that a number of mutations causing POLR3-related leukodystrophy impair proper assembly/biogenesis of Pol III [28, 30], often causing a retention of the unassembled subunits in the cytoplasm as revealed by immunofluorescence [30]. Unfortunately, drugs for the treatment of leukodystrophies have yet to be discovered.

Here, we report the development of an assay to dissect Pol III assembly. Our results show that Pol III assembly is a stepwise process in which interactions with the PAQosome and other cellular chaperones are observed. Most interestingly, we show that treatment with riluzole, an FDA-approved drug for amyotrophic lateral sclerosis (ALS) treatment [31, 32], partly corrects the defects in Pol III assembly in the presence of a POLR3B subunit having the R103H substitution known to be causative for POLR3-related leukodystrophy. Our results help to understand the mechanism of Pol III assembly and identify a potential therapeutic target for POLR3-related leukodystrophy.

## Results

### Pol III assembly is a stepwise process

**Fig. 1:**
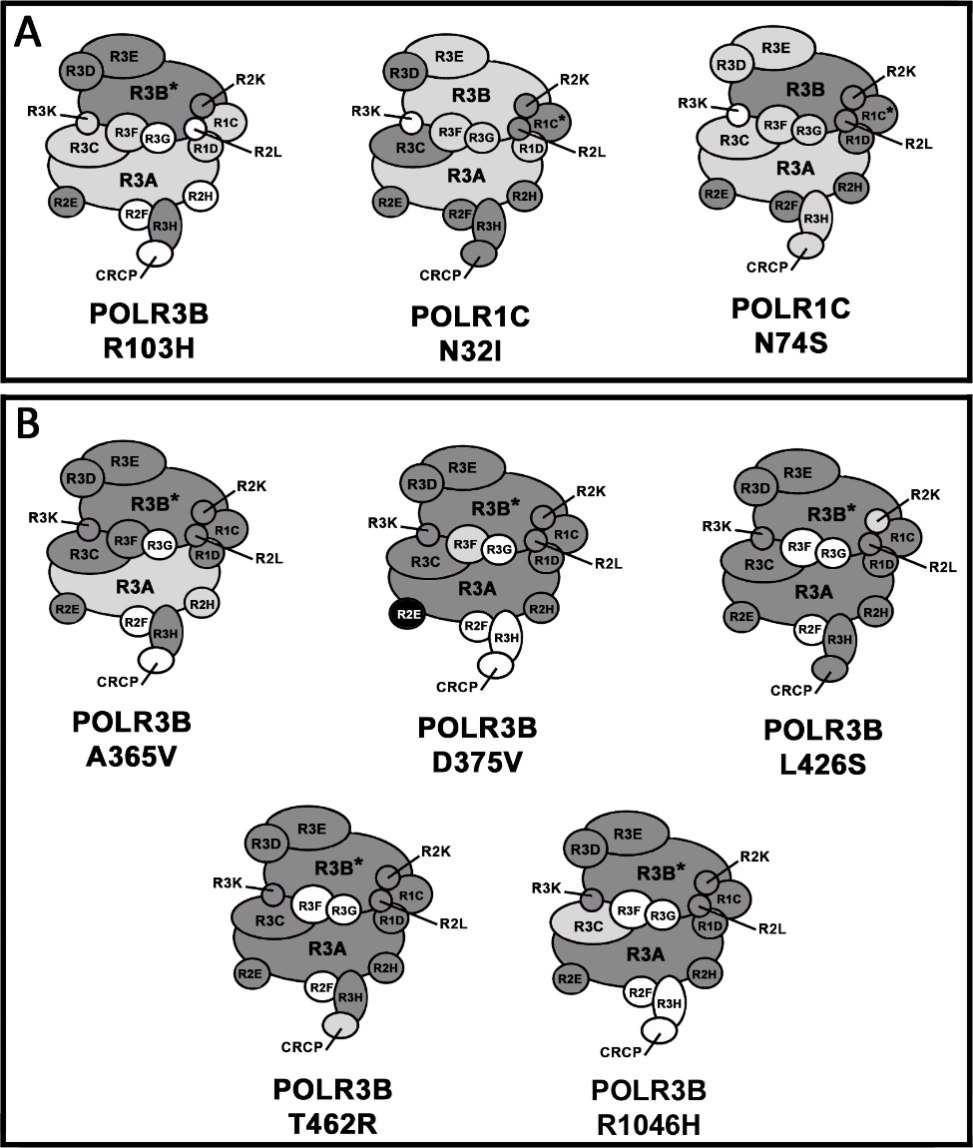
Schematic representation of Pol III assembly defects caused by various mutated subunits Representation of the 17-subunit Pol III (Pol III; reviewed in detail in 4) assembled in the presence of (**A**) the leukodystrophy-causative mutations encoding POLR3B-R103H, POLR1C-N32I and POLR1C-N74S and (**B**) the neurological disorder causative *de novo* mutations encoding POLR3B-A365V, POLR3B-D375V, POLR3B-L426S, POLR3B-T462R and POLR3B-R1046H. Asterisks (*) indicate the bait protein used in AP-MS. Light gray subunits indicate statistically significant reduction, black subunits indicate statistically significant increase and the white subunits were not detected in the AP-MS experiments. Dark gray subunits were detected but did not show statistically significant differences between the WT and the mutant

**Fig. 2:**
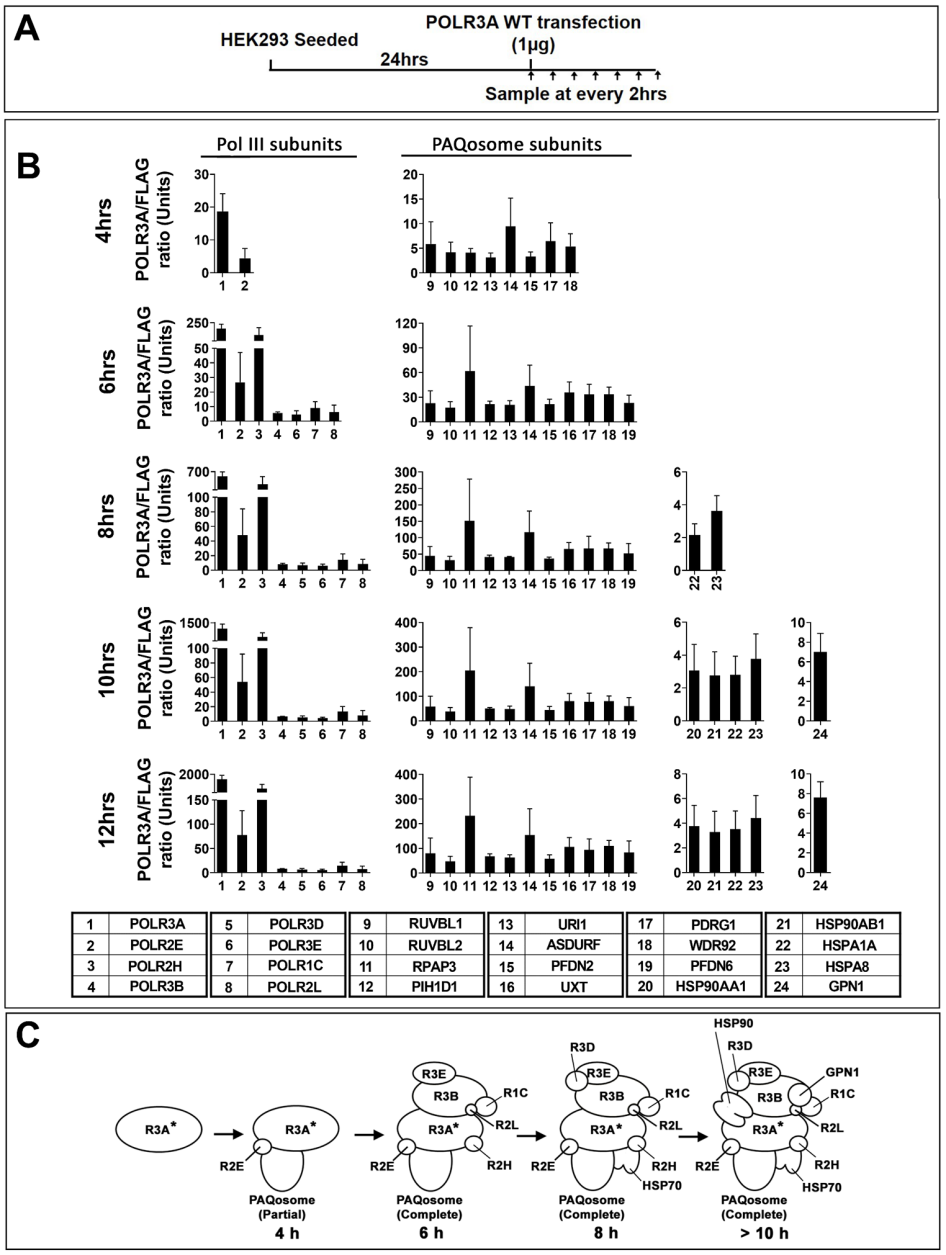
Time-course of Pol III assembly as determined by sequential AP-MS experiments using FLAG-POLR3A. FLAG-tagged POLR3A WT was transfected in HEK293 in triplicate and cells were harvested at time points indicated in the flow chart (**A**). Affinity purification was performed on 1.5 mg of cell extract for each time-point, dried, trypsin digested and quantified by LC-MS/MS. The LFQ intensity of each protein was computed using MaxQuant (version 1.6.17.0) against characterized Uniprot database (updated on June 3th 2018) and further analyzed by Perseus (Version 1.6.14.0) and GraphPad Prism 8 (Version 8.4.3). Proteins found in all triplicates were kept and Missing Not At Random (MNAR) values were imputed. Shown in (**B**) are histograms of indicated proteins significantly associated with POLR3A WT (n = 3). Proteins were deemed significantly associated when they had a False Discovery Rate (FDR) of p < 0.05 obtained via a two-tailed t-test adjusted with a permutation-based multiple hypothesis testing with 10,000 iterations and an s0 correction factor of 0.1 and the log_2_-transformed average LFQ-intensity difference between FLAG-Tagged POLR3A and the FLAG control ≥ 1. **C**) The model summarizes the multistep formation of the POLR3A subcomplex, following interaction between POLR3A subunit and 7 other Pol III subunits. Asterisks mark the subunits used as baits

In previous work, we have shown that Pol III subunits with specific amino acid substitution that cause leukodystrophy [28, 30] or other neurologic disorders [33] impair the assembly of the complete 17-subunit Pol III enzyme (Fig. 1) [28, 30, 33]. This conclusion is based on the results of multiple AP-MS experiments performed by pull-down of these transfected mutated subunits as compared to that of their WT counterparts. To better understand the molecular basis of these Pol III assembly defects, we sought to develop an assay that monitors subunit interactions that occur with various Pol III subunits through time. To do so, we analyzed by LC-MS/MS the affinity-purified FLAG-tagged POLR3A WT subunit from three independent experiments produced at various time points after its introduction in HEK293 cells by transient transfection as indicated in the flow chart in Fig. 2A. Pull-down of FLAG-tagged POLR3A WT showed statistically significant interaction with POLR2E at 4 h (Fig. 2B. and Table S1). This interaction was detected as soon as FLAG-tagged POLR3A WT expression was quantifiable by AP-MS (Fig. 2B and Table S1) or detected by Western blot (Fig. S1). Statistically significant interactions with POLR2H, POLR3B, POLR3E, POLR1C, and POLR2L were all observed at 6 h post-transfection. Finally, POLR3D showed statistically significant interaction at 8 h (Fig. 2B and Table S1). The 9 remaining Pol III subunits were not detected even in the latest time point tested or their level did not reach statistical difference as compared to the negative control. These results obtained by pulling-down the largest Pol III subunit indicate that Pol III assembly takes place in multiple steps as summarized in Fig. 2C.

### Multiple chaperones are specifically recruited during Pol III assembly

Pull-down of POLR3A WT also resulted in statistically significant purification of GPN1, the PAQosome and several HSPs during Pol III assembly (Fig. 2B and Table S1). More precisely, eight subunits of the PAQosome interacted with POLR3A at 4 h after transfection, whereas UXT, RPAP3 and PFDN6 were rather quantified at 6 h (Fig. 2B and Table S1). Moreover, our results show a statistically significant interaction with HSP70 and HSP90 at 8 and 10 h respectively (Fig. 2B and Table S1). GPN1, a co-factor that interacts with all three nuclear RNA polymerases [34], was significantly quantified at 10 h of the time course (Fig. 2B and Table S1). Our results indicate that several chaperones are recruited during Pol III assembly and that the PAQosome complex interacts rapidly with the POLR3A subcomplex, in a stepwise manner, not as a single 12-subunit pre-formed complex.

### Expression of the leukodystrophy-causative mutation POLR3B R103H leads to impaired Pol III complex assembly

**Fig. 3:**
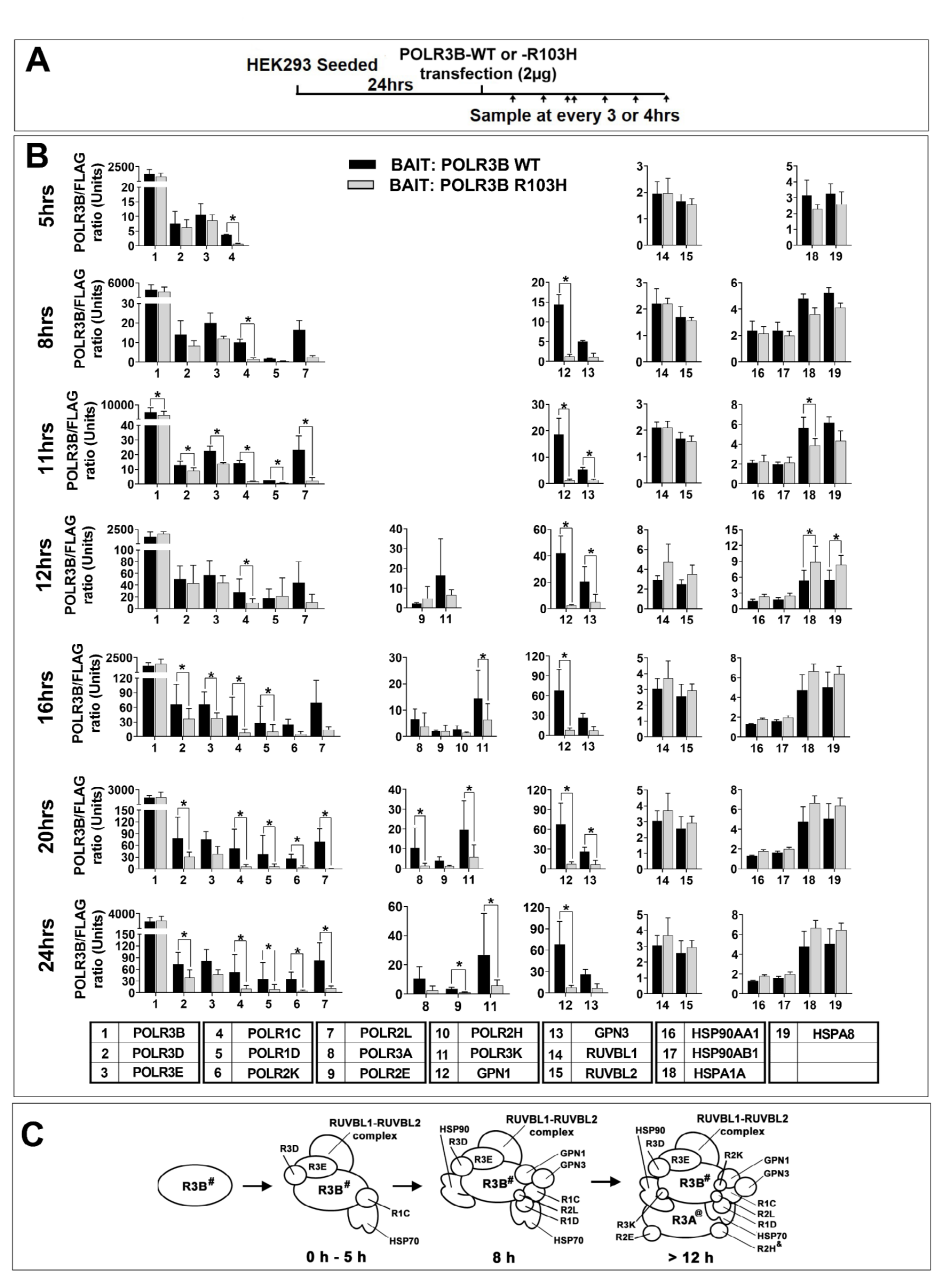
Impact of leukodystrophy-associated POLR3B R103H substitution on the time-course of Pol III assembly FLAG-tagged POLR3B WT or FLAG-tagged POLR3B R103H were transfected in HEK293 in triplicate and harvested at time points indicated in the flow chart (**A**). Affinity purification was performed as in Fig. 2. Shown in (**B**) are histogram of statistically significant association with POLR3B WT (n = 3) and POLR3B R103H (n = 3). POLR3B WT associated proteins were deemed statistically significant when they had a False discovery rate (FDR) of p < 0.05. **C**) The model summarizes POLR3B multi-step subcomplex formation following interaction between POLR3B and 10 other Pol III subunits. The #-marked subunit was used as bait. The @-marked subunit was statistically significant at 16 h and onward and the &-marked subunit was only statistically significant at 16 h

We previously reported that POLR3B with a R103H substitution impairs Pol III assembly as measured at 24 h after transfection [28]. Herein, we dissected the time-course of Pol III assembly both in FLAG-tagged POLR3B WT and FLAG-tagged POLR3B R103H, as indicated by the flow chart in Fig. 3A, in three independent experiments. As shown in Fig. 3B, POLR3D, POLR3E, and POLR1C showed statistically significant association with POLR3B WT at 5 h post transfection (Fig. 3B and Table S2) and POLR1D and POLR2L at 8 h. POLR3A, POLR2E, POLR2K, and POLR3K only interacted with WT POLR3B at 12 h and onward. These results demonstrate that, similarly to the POLR3A subcomplex, POLR3B subcomplex assembly occurs in multiple steps through time (summarized in Fig. 3C). Notably, all quantifiable subunits showed a statistically significant reduction of interaction with POLR3B R103H, as compared to WT, with POLR1C reduction being observable as early as 5 h post-transfection (Fig. 3B and Table S2). These results indicate that the assembly defects observed with POLR3B R103H occur early, during the initial step of complex assembly.

Similarly to when Pol III assembly was assessed using FLAG-POLR3A as a bait, we investigated the interactions between chaperones and POLR3B. GPN1 and GPN3 showed a statistically significant association with POLR3B WT at 8 h and this interaction was importantly reduced when the POLR3B R103H variant was used as the bait (Fig. 3B and Table S2). Notably, only the PAQosome subunits RUVBL1 and RUVBL2 showed statistically significant association with POLR3B (Fig. 3B and Table S2) as opposed to what was observed when POLR3A was pulled-down. In addition, the level of interaction between POLR3B and either RUVBL1 or RUVBL2 was not affected when FLAG-POLR3B R103H was used. We also observed a statistically significant interaction between POLR3B WT and various HSPs, namely, HSP90AA1, HSP90AB1, HSPA1A, and HSPA8. The interaction with HSP90AA1 and HSP90AB1 was not affected when FLAG-POLR3B R103H was used. However, a statistically significant reduction was observed at 11 h for HSP1A1 and at 12 h for HSPA8 but that difference disappeared after 16 h (Fig. 3B and Table S2). These results indicate that the POLR3B subcomplex rapidly interacts with various chaperones during complex assembly and that the R103H substitution affects only a subset of them.

### Riluzole can counteract Pol III assembly defects of POLR3B R103H

**Fig. 4:**
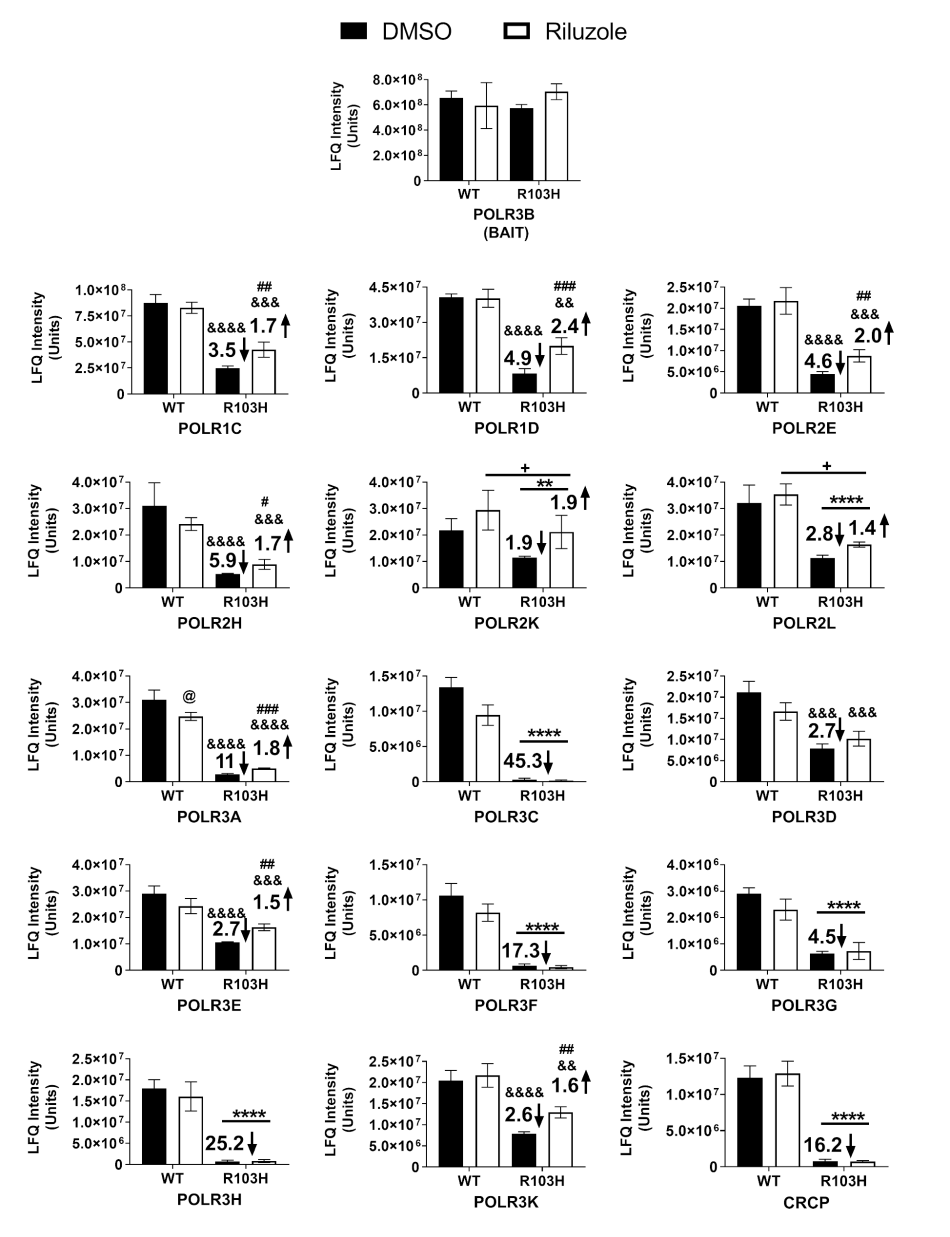
Riluzole treatment stimulates incorporation of the leukodystrophy-associated POLR3B R103H variant in Pol III. HEK293 cells transfected with FLAG-tagged POLR3B WT or R103H were treated with 12.5 µM of riluzole or vehicle (DMSO) for 20 h, in triplicate. Affinity purification was performed as in Fig. 2. Shown are the 17 POLR3 subunit mean LFQ intensities. Significant fold-decrease values (↓) against the DMSO treated WT and fold-increase values (↑) against the DMSO treated R103H values are indicated in each histogram. Two-way ANOVA statistical analysis was performed by using the log_2_-transformed imputated intensities. **: p < 0.01, ****: p < 0.0001; significant main effect of R103H mutation. +: p < 0.05; significant main effect of riluzole. #: p < 0.05, ##: p < 0.01, ###: p < 0.001; Significant effect of riluzole in R103H expressing cell. @: p < 0.05; significant effect of riluzole in WT expressing cell. &&: p < 0.01, &&&: p < 0.001, &&&&: p < 0.0001; significantly different that WT-DMSO.

A number of chemical compounds have previously been shown to affect proteostasis by targeting either the chaperone or proteasome machinery [35, 36]. We next sought to verify whether some of these characterized compounds can counteract the assembly defects (main effect) observed with FLAG-POLR3B R103H. Treatment of HEK293 cells expressing FLAG-tagged POLR3B R103H or WT with riluzole (IC_10_ = 12.5µM) (main effect), did not affect POLR3B expression levels (Fig. S2A) or other Pol III subunits (Fig. S2B). However, an interaction was found between riluzole treatment and cells expressing the leukodystrophy-related variant POLR3B R103H and this treatment stimulates Pol III assembly (Fig. 4, Fig. S3 and Table S3). As shown previously [28], Pol III subunits were significantly reduced in the FLAG-tagged POLR3B R103H samples as compared to WT (Fig. 4). The positive effect of riluzole was observed on both POLR3A and POLR3B subcomplexes (Fig. 4). Most of the subunits related to the POLR3B subcomplex were significantly increased by 1.5 to 2.4-fold (POLR1C, POLR1D and POLR3E) while POLR3A-subcomplex subunits were significantly increased by 1.7 to 2.0-fold (Fig. 4). These results indicate that riluzole can increase POLR3B R103H assembly in the Pol III complex.

## Discussion

POLR3-related leukodystrophy is a rare neurodegenerative disease and for which there is no cure. It affects previously healthy children and lead to progressive neurological symptoms, including motor and cognitive disturbances. Here, our approach has been to target the cause of this specific leukodystrophy. POLR3-related leukodystrophy is caused by biallelic pathogenic variants in genes encoding subunits of Pol III, i.e. POLR3A, POLR3B, POLR1C and POLR3K [25, 27–30, 37, 38]. AP-MS experiments revealed that some substitutions lead to subunits that cannot interact with their cognate partners to form a complete and functional Pol III enzyme [28, 30]. For example, POLR3B R103H causes a severe defect in complex assembly which makes it an interesting model to study Pol III assembly in cultured cells. In addition to its use as a model to study the molecular mechanism leading to leukodystrophy-associated assembly defects, it can be used as an assay for the discovery of drugs that act early, and directly on the cause of the disease. In this manuscript, we show that riluzole, an FDA-approved drug for the treatment of ALS, partially corrects Pol III assembly defects induced by the leukodystrophy-causative amino acid substitution R103H in POLR3B.

Assembly of Pol III was studied in yeast [39, 40] but to our knowledge, there is no report of its study in mammalian cells. Looking at various time points after transient expression of either FLAG-tagged WT POLR3A or POLR3B, we were able to purify and quantify the bait protein interacting with a number of other Pol III subunits and co-factors in a statistically significant manner. The models shown in Fig. 2C and Fig. 3 C summarize our data on the dynamics of Pol III assembly which shows striking similarities with Pol II complex formation as reviewed by Wild and Cramer [40]. In keeping with the Pol II model, we show that Pol III complex assembly follows a precise stepwise order that involves the formation of two distinct subcomplexes, each including one of the two largest Pol III subunits, POLR3A and POLR3B, and 9 other Pol III subunits. However, our approach cannot distinguish between the contribution of the various assembly stages that could exist at each time point. The importance of the different subunits in this stepwise process was further confirmed in POLR3B-subcomplex assembly when the variant POLR3B R103H was pulled-down. In this instance, the data show that the mutation affects complex formation early and that the lack of POLR3B-POLR1C interaction may be involved in this defect. This defect could be related to the POLR3B R103 amino acid environment. Pol III crystallography has shown that R103 is found in a negatively charged pocket close to the POLR2K and POLR2L interaction interface [4]. Histidine substitution would change the charge within the pocket and thus, cause protein instability affecting POLR2K, POLR2L and potentially POLR1C interaction with POLR3B. Other amino acid substitutions in this pocket region, namely L104F and R442C, were shown to be associated with POLR3-related leukodystrophy [4].

HSP90 and HSP70 have long been shown to participate in nascent protein folding, complex formation, and protein stability [41]. Moreover, the involvement of HSP90 in Pol II assembly [7, 40] requires the presence of the PAQosome core subunit RPAP3 [7]. Herein, we show that HSP70 and HSP90 also interact with POLR3A and POLR3B during Pol III assembly. However, their interaction with POLR3A was observed at later steps during this process, after 8-10 h. This either suggests that HSP70 and HSP90 act later to help in proper protein folding during Pol III assembly or that another chaperone such as the PAQosome [8] could be involved. On the other hand, early interaction with HSP70 followed by HSP90 was observed when POLR3B was pulled-down, suggesting that these chaperones do participate in POLR3B folding and subcomplex assembly.

Similarly to what has been reported for other RNA polymerases [6], we found that PAQosome subunits showed statistically significant association with the Pol III complex when POLR3A was pulled-down. This observation suggests that the PAQosome is involved in Pol III complex assembly. Inhibition of various PAQosome subunits has been shown to disrupt other protein complex formation such as that of Pol II [7], snRNP U5 [11] and PIKKs [19]. Our data further suggests that the interaction between POLR3A and the PAQosome occurs early, at the same time as POLR2E, a subunit that is common to both Pol III and PAQosome complexes. The strength of the interaction between POLR2E and URI shown in a previous study could explain this rapid recruitment of the PAQosome to POLR3A [42]. Interestingly, only two PAQosome subunits, namely RUVBL1 and RUVBL2, showed statistically significant association with the Pol III complex when POLR3B was pulled-down. Their interaction occurred early and was not affected by the leukodystrophy-associated substitution R103H in POLR3B. Further investigation is needed to determine the precise order of interactions between the POLR3A-subcomplex and PAQosome subunits, to determine its function in the proper assembly of Pol III complex and to increase our understanding of the exact role of the PAQosome in the process.

GPN1 and GPN3 were also found to significantly interact with the POLR3B subcomplex. These two co-factors are essential for the biogenesis of Pol II [43] and were shown to be required for nuclear translocation of Pol II and Pol III [44]. Niesser et al. have shown that they can act as GTPase-driving chaperones [43]. Interestingly, GPN1 and GPN3 were recruited with the POLR3B subcomplex and these interactions were significantly reduced in the R103H variant, suggesting that POLR1C, and potentially other interacting subunits such as POLR1D and POLR2L, are required for GPNs’ recruitment at the subcomplex. This hypothesis is supported by the fact that GPN1 was significantly reduced in the POLR1C N74S variant as observed by Thiffault et al. [30]. Moreover, the amino acid N74 was shown by Ramsay et al. [4] and Girbig et al. [5] to be located at an interface that mediates POLR1C interaction with POLR3B [4, 5]. Further investigation is required to understand the exact role of GPNs during Pol III assembly.

Proteostasis is a complex process involving various systems such as chaperones and the proteasomes that can be used as therapeutic targets [45, 46]. Various compounds targeting these systems have proven efficacious at improving folding of several key proteins associated with neurological diseases [47]. One of these compounds, riluzole, has been shown to act as an antiglutamate drug that blocks excessive release of glutamate in motor neurons [48, 49], inhibiting Na^+^ channel [50] in ALS animal models, and has since been the subject of repurposing studies for various neurological diseases [51] and cancers [52, 53]. In our experiments, riluzole treatment resulted in a positive impact on Pol III assembly in the leukodystrophy-causative POLR3B R103H model. Specifically, riluzole was able to significantly increase the interaction level between POLR1C and POLR3B as well as other Pol III subunits. Even if the impact of the R103H variant on small RNAs expression level is not known, it was shown that complex assembly could affect expression level of some small RNA in other Pol III complex assembly defects, as we have previously shown with POLR1C variants [30]. Further experiments would be required to investigate if riluzole could rescue R103H-expressing Pol III function. The exact mechanism of action of riluzole in our model and whether it is beneficial for other leukodystrophy-causative mutations with assembly defects remains unknown. However, Yang et al. have shown that riluzole can affect the expression level of HSF1 in the cytoplasm [54] and its translocation stimulates the expression of proteostasis-associated molecules [55]. Work in progress will precise the mechanism of action of riluzole, particularly if the PAQosome or its expression is the target of its action on Pol III assembly. But at this stage, this is purely speculative.

## Conclusion

Our results define in further details the mechanism of Pol III assembly, confirm an extensive association of the PAQosome during the process, and identify an FDA-approved drug, riluzole, that now needs to be further studied to assess its usefulness to treat POLR3-related leukodystrophy caused by assembly defects.

## Materials and methods

### Cell culture and drug treatment

Human embryonic kidney cell line 293 (HEK293) were maintained in culture in DMEM containing 4.5 g/L glucose (ThermoFisher, 11995-065) supplemented with 10% fetal bovine serum (Wisent, 080–150), 2 mM glutamine (ThermoFisher, 25,030,081), 100 U/mL penicillin and 100 µg/mL streptomycin (ThermoFisher, 15,140,122). To obtain similar expression dynamics in HEK293, 4.26 µg of FLAG-tagged POLR3A WT or 8.51 µg of FLAG-tagged POLR3B (WT or R103H) expression vectors produced previously [26, 28] were transiently transfected in 15 cm plates containing 1.6 × 10^7^ cells/plate, grown overnight, by using Jet Prime transfection reagent (PolyPlus) according to manufacturer’s instruction. Transfected cells were incubated at 37 °C for indicated time, harvested on ice, washes with iced-cold 1X PBS and snap freeze in liquid nitrogen. In indicated experiments, 12.5 µM of riluzole (Sigma-Aldrich, R115-25MG) was added 4 h after transfection of either the WT, the R103H mutant of FLAG-tagged POLR3B and the emptied vector and transfected cells were harvested 20 h later on ice. DMSO-treated transfected cells were used as negative controls.

### FLAG affinity purification

This protocol was modified from Kean et al. [56]. Cell membranes were disrupted by a 30 min incubation at 4 °C on a tube rotator followed by a liquid nitrogen freeze-thaw cycles in lysis buffer (25 mM HEPES, 1 mM EDTA, 0,05% NP-40, 5% Glycerol, 50 mM KCl, 1 mM DTT, 1X cOmplete EDTA-free Protease Inhibitor Cocktail (Millipore Sigma, COEDTAF-RO), 1 mM PMSF, 10 mM NaF and 1 mM Na_3_VO_4_). The protein extracts were cleared of insoluble material by centrifugation (16 000 xg, 30 min, 4 °C) and the protein content was assessed by Bradford assay. 30 µL of anti-FLAG (M2) magnetic bead slurry (MilliporeSigma, M8823) were washed four times with 1 mL of lysis buffer, then incubated for three hours with 1.5 mg of total proteins extract at 4 °C on a tube rotator. The beads were then washed four times in 1 mL of lysis buffer and four more times in 1 mL wash buffer (75 mM KCl, 50 mM ammonium bicarbonate pH 8.0). The bound proteins were eluted by three successive 15 min incubations in 150 µL of ammonium hydroxide solution pH 11–12 (roughly 10%) in HPLC-grade water (Sigma, 7732-18-5). The combined fractions were dried in a speed-vac and then resuspended in 10 µL of 6 M urea. Reduction buffer was added at a volume of 2.5 µL (45 mM DTT, 100 mM ammonium bicarbonate) and the samples were incubated for 30 min at 37 °C. An additional 2.5 µL of alkylation buffer (100 mM iodoacetamide, 100 mM ammonium bicarbonate) were included to the mix followed by 20 min incubation at 24 °C and in absence on light. 20 µL of HPLC-grade water were added to reduce urea concentration and trypsin digestion was performed using a 1:20 (enzyme:protein) ratio of sequencing grade modified trypsin (Promega, V5111) and 18 h incubation at 37 °C on a ThermoMixer (Eppendorf, 5,382,000,023). Following a quick centrifugation (500 xg, 1 min) the peptides were collected. Trifluoroacetic acid was added to the samples and residual salts and detergents were removed using an Oasis MCX 96-well Elution Plate (Waters, 186001830BA) loaded onto a Positive Pressure-96 Processor (Waters, 186,006,961) according to the manufacturer’s instructions. Eluates were dried down in vacuum centrifuge and then re-solubilized under agitation for 15 min in 11 µL of 2% acetonitrile, 1% formic acid, of which 5 µL were used for LC-MS/MS.

### Mass spectrometry

High performance liquid chromatography (HPLC) was performed using C18 resin (5 μm particles, 300 Å pores) extracted from a Jupiter LC Column (00B-4053-E0) and packed in a PicoFrit Column (NEW OBJECTIVE, PF360-75-15-N-5), the latter of which was loaded into a Easy-nLC II instrument (ThermoFisher). Employed buffers were: 0.2% formic acid (buffer A) and 100% acetonitrile/0.2% formic acid (buffer B). Peptide elution was performed with a two-slope gradient at a flowrate of 250 nL/min. Solvent B gradually increased from 2 to 37% over the course of 90 min and then from 37 to 80% B in 10 min. The HPLC system was coupled to an Orbitrap Fusion Tribrid mass spectrometer (Thermo Scientific, IQLAAEGAAPFADBMBCX) through a nano-ESI source (ThermoFisher, ES071). Nanospray and S-lens voltages were set to 1.3–1.8 kV and 50 V, respectively. Capillary temperature was set to 225 °C. Full scan MS survey spectra (m/z 360–1560) in profile mode were acquired in the Orbitrap with a resolution of 120,000 with a target value at 1e6. The 25 most intense peptide ions were fragmented in the HCD collision cell and analyzed in the linear ion trap with a target value at 2e4 and normalized collision energy at 28. Target ions selected for fragmentation were dynamically excluded for 25 s.

### Data analysis

Label-Free Quantification (LFQ) intensity for each proteins were obtained by using MaxQuant (version 1.6.17.0) [57] against the characterized human UniProtKB database (release on June 3th 2018). Log2 transformation, imputation and further statistical analysis were performed with Perseus (version 1.6.14.0) [58] or Graphpad Prism 8 (Version 8.4.3). All purifications were done in triplicate and proteins detected in all experiments were kept for further analysis. Missing values were replaced by randomly generated intensities normally distributed with a width of 0.3 times and a downshift of 1.8 times the standard deviation of non-zero intensities. Significant differences between Log2 protein intensities from bait purifications and corresponding control groups were then determined using a two-tailed T-test subsequently adjusted for multiple hypothesis testing with a permutation-based False Discovery Rate (FDR) of 0.05 and a fudge factor (s0) of 0.1 with 10,000 iterations. For WT and mutant comparison or riluzole- and DMSO-treated samples comparison, proteins that did not show enrichment in either conditions compared to their respective control (i.e. p-values > 0.05 and ratio ≤ 2) were discarded. In appropriate experiments, Log2-transformed LFQ-intensities were analyzed using a Two-way ANOVA. In the event of a significant probability value, Tukey post-hoc multiple comparison test was used in drug-treated WT or mutant samples. For all analyses, p ≤ 0.05 was considered statistically significant.

## Electronic supplementary material

Below is the link to the electronic supplementary material.

Supplementary Material 1: **Table S1**. Filtered proteins list from MaxQuant imputated label-free quantification intensity analysis of POLR3A_0–12 h time course experiment related to Fig. 2

Supplementary Material 2: **Table S2**. Filtered proteins list from MaxQuant imputated label-free quantification intensity analysis of POLR3B_5–11 h and 12–24 h time course experiment related to Fig. 3

Supplementary Material 3: **Table S3**. Filtered protein list from MaxQuant label-free quantification analysis of Riluzole treat POLR3B-WT and mutant experiment related to Fig. 2 A and 2B. Experiment related to supplementary Fig. S3A and S3B

Supplementary Material 4: **Figure S1**: POLR3A WT expression is rapidly detected in transiently transfected cells. **Figure S2**: Riluzole treatment does not affect POLR3B WT, R103H or other Pol III subunit expression levels. **Figure S3**: Riluzole treatment increases incorporation of POLR3B R103H.

## Data Availability

The mass spectrometry proteomics data have been deposited to the ProteomeXchange Consortium via the PRIDE [59] partner repository with the dataset identifier PXD034961.

## Abbreviations

ALS: Amyotrophic lateral sclerosis
AP-MS: Affinity purification coupled to mass spectrometry
ATM: Ataxia-telangiectasia mutated
ATR: ATM and Rad3-related
cDNA: Complementary DNA
DMEM: Dulbecco’s Modified Eagle Medium
DMSO: Dimethyl Sulfoxide
DTT: 1,4-Dithiothreitol
FDA: Food and Drug Administration
FDR: False Discovery Rate
GPN: GPN-loop GTPase
HLD: Hypomyelinating leukodystrophy
HEK293: Human embryonic kidney cell line 293
HPLC: High performance liquid chromatography
HSP: Heat Shock Protein
MRI: Magnetic resonance imaging
LFQ: Label-Free Quantification
PAQosome: Particle for Arrangement of Qarternary structure
PFDL: Prefoldin-like
PIKK: Phosphatidylinositol 3-Kinase-related kinases
Pol III: RNA Polymerase III
rRNA: ribosomal RNA
SMG-1: Upf1 complex
snRNA: Small-nuclear RNA
tRNA: transfer RNA
WT: Wild-type
