# Supplementary material for: Riluzole partially restores RNA polymerase III complex assembly in cells expressing the leukodystrophy-causative variant POLR3B R103H
Source: Mol Brain. 2022 Nov 30;15:98. doi: 10.1186/s13041-022-00974-z (PMC9710144; doi:10.1186/s13041-022-00974-z)
Supplement: Supplementary file 4 — Supplementary Material 4: Figure S1: POLR3A WT expression is rapidly detected in transiently transfected cells. Figure S2: Riluzole treatment does not affect POLR3B WT, R103H or other Pol III subunit expression levels. Figure S3: Riluzole treatment increases incorporation of POLR3B R103H. [file 13041_2022_974_MOESM4_ESM.docx]

**Supplementary Figure legends**


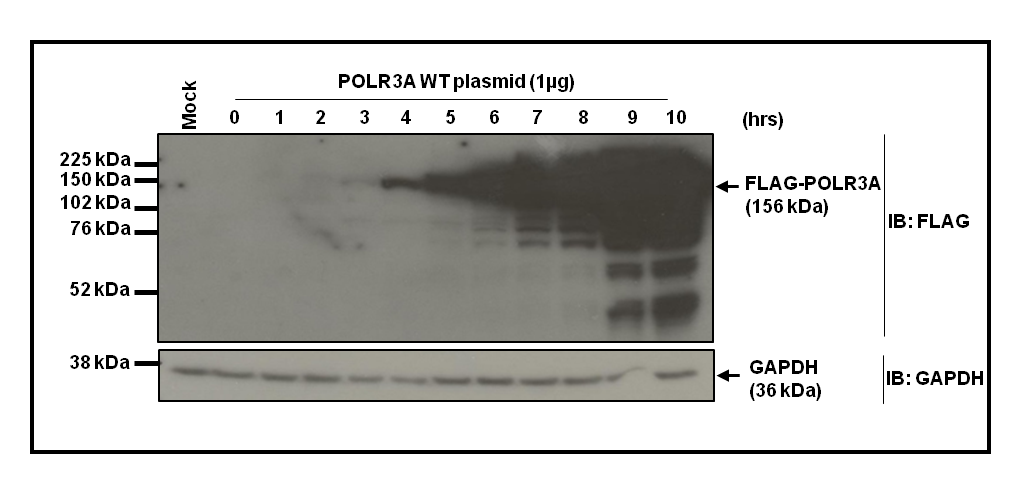


**Supplementary Figure 1: POLR3A WT expression is rapidly detected in transiently transfected cells.**

Whole cell extracts were obtained from FLAG-tagged POLR3A WT transfected HEK293 cells harvested and lysed at indicated times. FLAG-tagged-POLR3A expression level was assessed by western blot with an anti-FLAG (M2) primary antibody (1:2000). GAPDH (1:1000) was used as loading control.


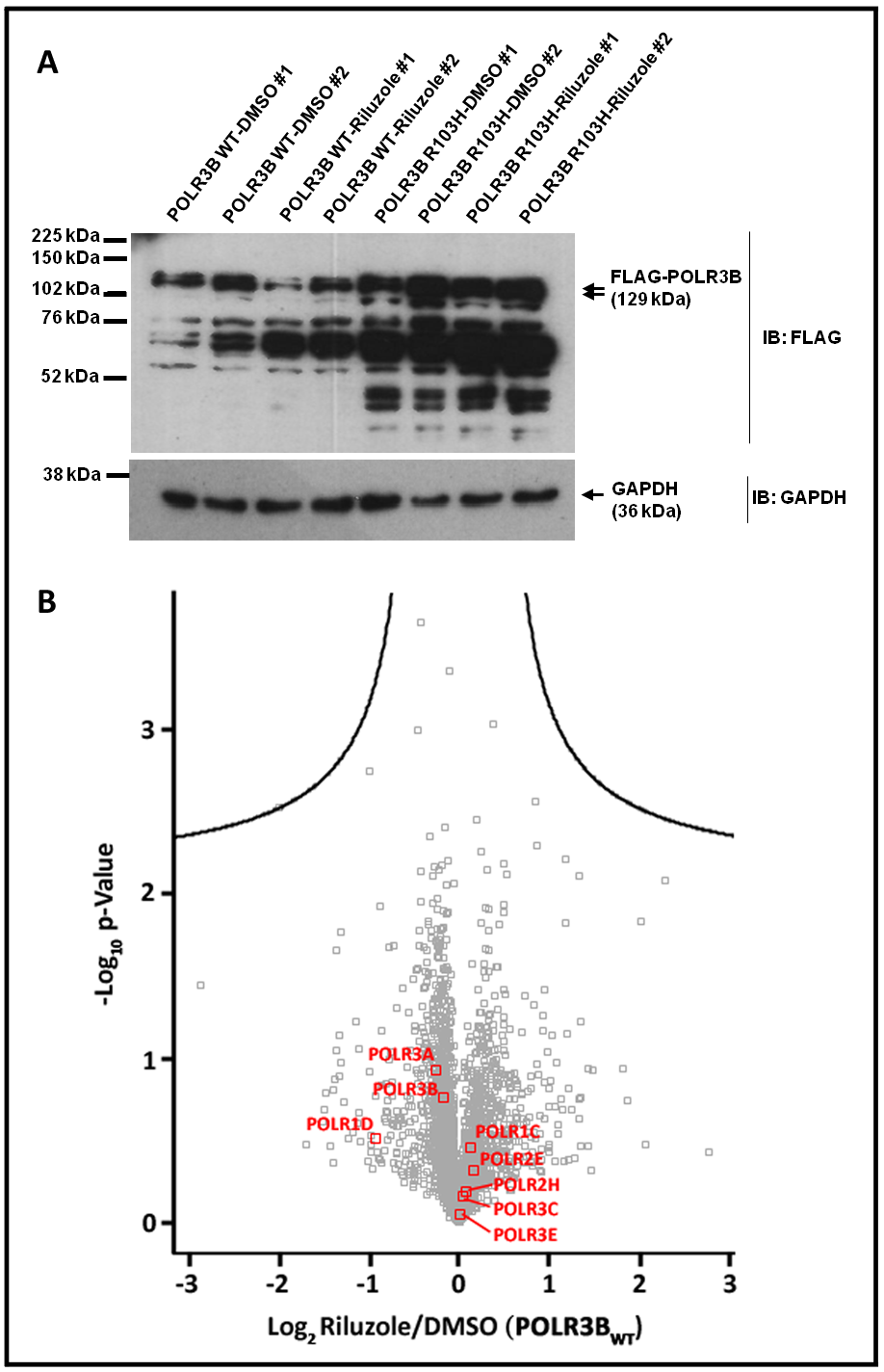


**Supplementary Figure 2: Riluzole treatment does not affect POLR3B WT, R103H or other Pol III subunit expression levels.**

A) Whole cell extracts were obtained from FLAG-tagged POLR3B WT or R103H transfected cell treated with 12.5 µM of riluzole or vehicle (DMSO) for 20 h, in duplicate. FLAG-tagged-POLR3B expression level was assessed by western blot with an anti-FLAG (M2) primary antibody (1:2000). GAPDH (1:1000) was used as loading control. B) HEK293 cells transfected with FLAG-tagged POLR3B WT treated with 12.5 µM of riluzole or vehicle (DMSO) for 20 h, in triplicate. Whole cell extracts were acetone-precipitated, dried, trypsin digested and quantified by LC-MS/MS. The LFQ intensity of each protein was computed via MaxQuant (version 1.6.17.0) against characterized Uniprot database (updated on June 3th 2018) and further analyzed with Perseus (Version 1.6.1.3). The volcano plot presents the log_2_-transformed average LFQ-intensity difference between the Riluzole-treated WT against the control DMSO-treated WT (x-axis). –log_10_ p value obtained via a two-tailed t-test adjusted with a permutation-based multiple hypothesis testing with 10,000 iterations and an s0 correction factor of 0.1 (y axis). Detected Pol III subunits are marked in red. No proteins were significantly different.


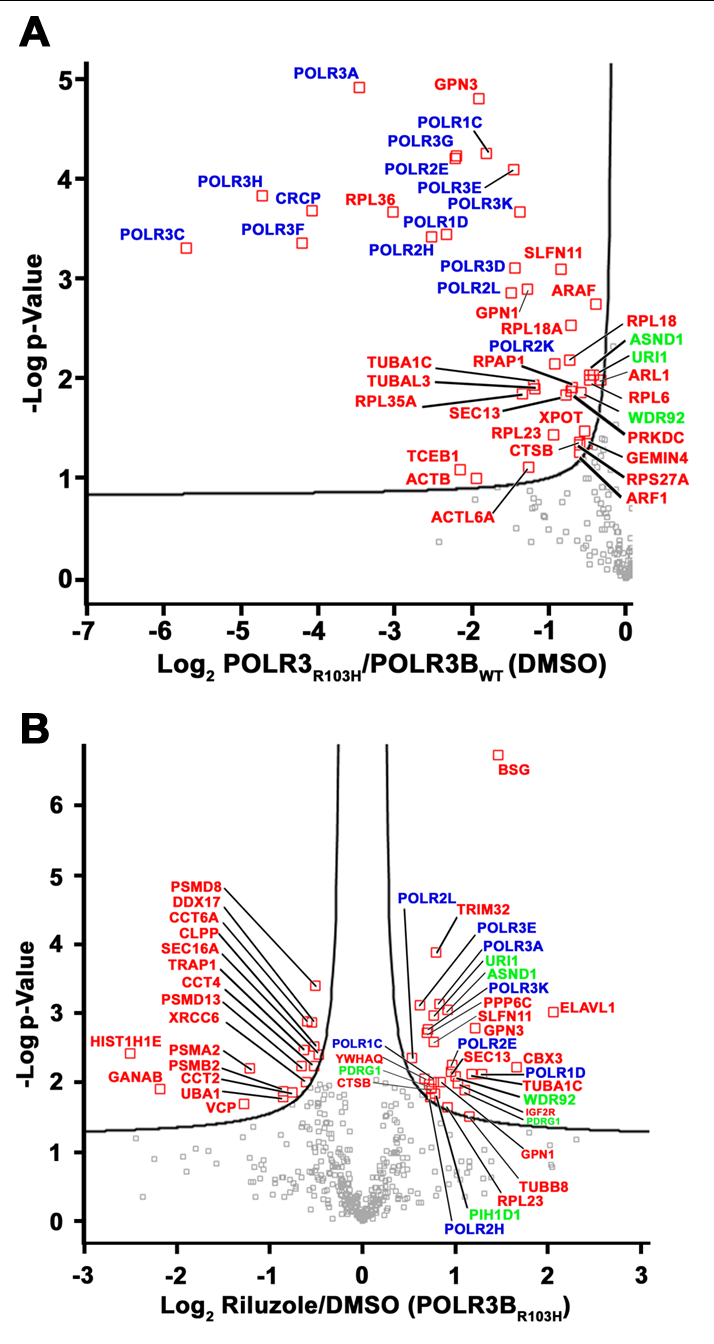


**Supplementary Figure 3: Riluzole treatment increases incorporation of POLR3 R103H.**

HEK293 cells transfected with FLAG-tagged POLR3B WT or R103H were treated with 12.5 µM of riluzole or vehicle (DMSO) for 20 h, in triplicate. Affinity purification was performed on 1.5 mg of cell extract for each experimental conditions, dried, trypsin digested and quantified by LC-MS/MS. The LFQ intensity of each protein was computed via MaxQuant (version 1.6.17.0) against characterized Uniprot database (updated on June 3th 2018) and further analyzed with Perseus (Version 1.6.1.3). Shown volcano plots illustrate the log_2_-transformed average LFQ-intensity difference between (A) the DMSO-treated R103H mutant against the DMSO-treated WT or (B) the riluzole treated R013H mutant against his DMSO-treated control (x-axis). –log_10_ p value obtained via a two-tailed t-test adjusted with a permutation-based multiple hypothesis testing with 10,000 iterations and an s0 correction factor of 0.1 (y axis). The significantly different Pol III subunits were marked in blue, the significantly different PAQosome subunits were marked in green and the proteins marked in red are considered significantly different between the conditions.
